# Recombinant Promoter (MUASCsV8CP) Driven Totiviral Killer Protein 4 (KP4) Imparts Resistance Against Fungal Pathogens in Transgenic Tobacco

**DOI:** 10.3389/fpls.2018.00278

**Published:** 2018-03-05

**Authors:** Debasish Deb, Ankita Shrestha, Indu B. Maiti, Nrisingha Dey

**Affiliations:** ^1^Division of Plant and Microbial Biotechnology, Institute of Life Sciences, Bhubaneswar, India; ^2^Department of Molecular Plant Virology and Plant Genetic Engineering, KTRDC, College of Agriculture, Food and Environment, University of Kentucky, Lexington, KY, United States

**Keywords:** killer protein, *Alternaria alternata*, *Phoma exigua*, caulimovirus, recombinant promoter

## Abstract

Development of disease-resistant plant varieties achieved by engineering anti-microbial transgenes under the control of strong promoters can suffice the inhibition of pathogen growth and simultaneously ensure enhanced crop production. For evaluating the prospect of such strong promoters, we comprehensively characterized the full-length transcript promoter of *Cassava Vein Mosaic Virus* (CsVMV; -565 to +166) and identified CsVMV8 (-215 to +166) as the highest expressing fragment in both transient and transgenic assays. Further, we designed a new chimeric promoter ‘MUASCsV8CP’ through inter-molecular hybridization among the upstream activation sequence (UAS) of *Mirabilis Mosaic Virus* (MMV; -297 to -38) and CsVMV8, as the core promoter (CP). The MUASCsV8CP was found to be ∼2.2 and ∼2.4 times stronger than the CsVMV8 and CaMV35S promoters, respectively, while its activity was found to be equivalent to that of the CaMV35S^2^ promoter. Furthermore, we generated transgenic tobacco plants expressing the totiviral *‘Killer protein KP4’ (KP4)* under the control of the MUASCsV8CP promoter. Recombinant KP4 was found to accumulate both in the cytoplasm and apoplast of plant cells. The agar-based killing zone assays revealed enhanced resistance of plant-derived KP4 against two deuteromycetous foliar pathogenic fungi viz. *Alternaria alternata* and *Phoma exigua* var. *exigua*. Also, transgenic plants expressing *KP4* inhibited the growth progression of these fungi and conferred significant fungal resistance in detached-leaf and whole plant assays. Taken together, we establish the potential of engineering “in-built” fungal stress-tolerance in plants by expressing *KP4* under a novel chimeric caulimoviral promoter in a transgenic approach.

## Introduction

Designing of pathogen-resistant plants can help to alleviate global food crisis. An annual crop loss of 10% occurs worldwide due to fungal diseases; fungicides are the most commonly used means to prevent the colonization, sporulation, and growth of phytopathogenic fungi (Hahn, 2014; Drenth and Guest, 2016). But, the situation is worsened by the appearance of resistant isolates of various pathogens, which render the regular use of these fungicides, impractical (Nicot et al., 2016). In addition, the use of potent fungicides has already been pronounced as ‘obsolete’ due to the presence of high genetic diversity of phytopathogens as well as undesirable after-effects of these chemicals on the environment (Burdon, 1993; Damalas and Eleftherohorinos, 2011; Lorenzini and Zapparoli, 2014; Czislowski et al., 2017). There are numerous resistant varieties of plant fungal pathogens that have been documented viz. *Colletotrichum graminicola* causing anthracnose of turf grasses (Avila-Adame et al., 2003), *Plasmopara viticola* causing downy mildew of grapes (Genet et al., 2006), *Venturia inaequalis* causing apple scab (Köller et al., 2004), *Monilinia fructicola* causing brown rot of stone fruits (Chen et al., 2013), *Botrytis cinerea* causing post-harvest gray mold of apple (Hahn, 2014) and many other. In continuation, plant-pathogenic species of *Alternaria* cause devastating effects on crops of nutritional and economic value by attacking several members of *Cucurbitaceae*, *Brassicaceae*, and *Solanaceae* plant families (Mamgain et al., 2013). *Phoma*, on the other hand, causes epidemic disease development in cabbage, Tasmanian pyrethrum and oilseed rape (West et al., 2001; Pethybridge et al., 2005; Dilmaghani et al., 2009). Besides, it has devastating effects on field peas when associated with *Ascochyta* (Liu et al., 2016). Recently, both *Alternaria* and *Phoma* were found to develop resistance against QoI or strobilurins and benzimidazole group of fungicides, respectively (Van de Graaf et al., 2003; Karaoglanidis et al., 2011; Kim et al., 2017). *Alternaria* directly penetrates and infects annual plants including several vegetables, ornamentals and fruit trees (Sharma et al., 2013). On the other-hand, *Phoma* being a hemibiotrophic pathogen enters through wounds and attacks several herbaceous plants including vegetables like common bean, artichoke, romaine lettuce, and field pea (Kubota and Abiko, 2002; Fitt et al., 2006; Koike et al., 2006; Khani et al., 2016). Furthermore, *Phoma-Didymella* complex in association with *Peronospora sparsa* causes epidemic wilting symptom in Arctic bramble (*Rubus arcticus* L.) (Lindqvist-Kreuze et al., 2003) in Finland. On one-hand, where such pathogens cause heavy crop losses, there are serious constraints associated with the emerging resistant strains of the same. Therefore, there is an urgent need to explore new alternatives that can mount a broader resistance spectrum as well as stable pattern of inheritance in the resistant plants (Russell, 2013).

Till date there have been numerous advances toward the development of disease-resistant plant varieties via conventional breeding (Collard and Mackill, 2008). However, raising biotic-stress tolerant plants by traditional breeding approaches is fairly slow and may lead to unwanted linkage-drag due to the presence of quantitative traits at multiple loci (Jauhar, 2006). In contrast, the transgenic approach offers an excellent asexual means to insert foreign genes into plant cells (Sanghera et al., 2011).

The need to develop proficient plant-expression systems coupled to novel regulatory elements (promoter) that can efficiently escalate the disease resistance by expressing anti-microbial transgenes (at moderate/high level) in the engineered plants is gradually increasing (Rushton et al., 2002). Usually, the naturally occurring promoters have certain limitations and have low transcriptional activities. On the contrary, recombinant synthetic promoters are generated through shuffling of unique domains namely the distal upstream activation sequence (UAS) and proximal core promoter (CP) region of the same or different naturally occurring promoters. Such tailor-made chimeric promoters with altered *cis*-architecture usually appear to be efficient in driving transgenes in a plant cell (Dey et al., 2015). For example, the M24 promoter (Dey and Maiti, 1999), a chimera containing the duplicated enhancer domains derived from the *Mirabilis Mosaic Virus* (MMV) is capable of driving constitutive expression of heterologous genes in engineered plants such as, the SAC domain of *PAR-4* (*Prostate Apoptosis Response 4*) (Sarkar et al., 2015) and *Glucocerebrosidase (GCB)* (Chatterjee et al., 2017). The chimeric promoter ‘MUAS35SCP’ (Patro et al., 2012) has been established to drive constitutive and stable expression of human beta-defensins (*hβD-1 and hβD-2*) in transgenic tobacco lines (Patro et al., 2015). Likewise, in the present study, we generated a chimeric promoter “MUASCsV8CP” by coupling the UAS of MMV (MUAS) with the newly identified CsVMV8 promoter fragment of *Cassava vein mosaic virus* (CsVMV), as CP and tested its efficacy in plant modification. The CsVMV, a putative member of the caulimoviral group infects cassava (*Manihot esculenta* L.) plants in Brazil. It has a unique genome organization with a promoter sequence that contains several organ-specific *cis*- elements and has been partially characterized in rice and cassava (Verdaguer et al., 1998; Oyelakin et al., 2015). In this context, the transcription start site (TSS) of CsVMV promoter was earlier reported as an adenine residue located 35 bp downstream of the TATA-box (Verdaguer et al., 1996).

Transgenic plants expressing anti-fungal proteins have previously been tested for disease tolerance. The first fungus-resistant transgenic plant expressing bean-chitinase in tobacco and *Brassica napus* conferred resistance toward *Rhizoctonia solani* (Broglie et al., 1991). Since then, various transgenic plants such as those expressing Glucose *oxidase* gene from *Talaromyces flavus* (Murray et al., 1997), family 19 chitinase of *Streptomyces griseus* (Itoh et al., 2003), endochitinase gene from *Trichoderma virens* (Emani et al., 2003), bean chitinase from *Phaseolus vulgaris* (Tohidfar et al., 2005) and *Rpsl-k* from Soyabean (Gao et al., 2005) have provided increased resistance to various fungal pathogens. The corn smut fungus, *Ustilago maydis* secretes a virally encoded fungal toxin “Killer Protein 4” (KP4) when infected by the P4 strain of UMV4 totivirus (Allen et al., 2011). KP4 killer toxin is a α/β sandwich protein having 105 amino acids and is the only member of Killer protein family that is not processed by Kex2p (Ganesa et al., 1991). KP4 functions as a calcium channel chelator that blocks a cAMP-regulated growth pathway (Gage et al., 2001). In continuation, Schlaich et al. (2006) reported that KP4 specifically blocks the calcium channels of fungi. However, it has no effect on human and plants. Furthermore, transgenic maize expressing *KP4* was found to be highly resistant to corn smut, a devastating fungal disease where galls start appearing on aboveground parts of plants particularly on leaves. This calls for a comprehensive evaluation of the anti-fungal properties of KP4 wherein we can find other candidate fungi that can be targeted using this molecule in a transgenic approach.

In this study, we fully characterized the full-length transcript promoter of CsVMV. We were able to identify the ‘CsVMV8’ promoter having equivalent activity to the most widely used CaMV35S promoter. To further enhance the activity of CsVMV8, we performed inter-molecular hybridization between the MUAS and CsVMV8-CP (CsV8CP) to generate a chimera ‘MUASCsV8CP’. The activity of this newly designed chimeric promoter (coupled to a GUS reporter) was evaluated in the transient (protoplast and agro-infiltration) and transgenic assays. Further, this recombinant promoter was employed for raising transgenic tobacco lines expressing *KP4*. Next, we examined the fungal resistance of transgenic lines expressing KP4 against two foliar pathogenic fungi namely *Alternaria alternata* and *Phoma exigua* var. *exigua*. The *in vitro* agar-based killing zone and leaf detachment assays were performed to evaluate whether the recombinant KP4 could substantially restrain the growth of both the above fungi. To further inspect the anti-fungal activity of KP4 transgenic plants, we performed the disease resistance assays using whole plants against them. Overall, this study describes the effectiveness of the totiviral *KP4* driven by a newly designed chimeric caulimoviral promoter MUASCsV8CP to restrict the growth of devastating foliar fungi, which may open new avenues in the field of plant protection against phyto-pathogens.

## Materials and Methods

### Construction of Binary Vectors for Expression in Plants

A total of thirteen 5′- (CsVMV1-CsVMV13) and three 3′- (CsVMVR1-CsVMVR3) end deletion fragments were PCR amplified (using primers listed in **Supplementary Table S1**) adding *EcoR*I and *Hind*III sites at the 5′- and 3′- ends, respectively. The PCR amplified products were subsequently cloned in the *EcoR*I and *Hind*III sites of pUC119 sequencing vector and subsequently sequenced and analyzed to check their integrity. The fragments were finally sub-cloned into protoplast and plant expression vectors pUCPMAGUS and pKYLX71GUS (Schardl et al., 1987; Dey and Maiti, 1999), respectively.

For the generation of chimeric promoter construct MUASCsV8CP, the UAS of MMV (MUAS; 335 bp, -297 to -38) and the proximal domain of CsVMV8-Flt (CsV8CP; 381 bp, -215 to +166) promoter were PCR amplified using appropriate primers (**Supplementary Table S1**) containing overhangs of *EcoR*I and *Hinc*II at the 5′- end and *Sma*I and *Hind*III at the 3′- end. The PCR amplified fragments were cloned into the corresponding sites of pUC119 following a previously published protocol (Acharya et al., 2014). Subsequently, the resultant promoter fragment was sub-cloned into pUCPMAGUS and pKYLX71GUS to generate the pUPMUASCsV8CPGUS and pKMUASCsV8CPGUS plant expression vectors.

To generate the recombinant *KP4* chimeric construct, the totiviral *KP4* gene [UniProtKB-Q90121(KP4T_UMV4)] was fused with a 35-nucleotide untranslated region of AlMV RNA4 (5′ AMV; translational enhancer) and apoplast targeting sequence (aTP) of *Arabidopsis* 2S2 protein gene (Kroumova et al., 2013) at its 5′- end; and a hexa (6x) His-tag at its 3′- end. The above recombinant sequence was then cloned into the *Xho*I and *Sst*I sites of the binary vector pKMUASCsV8CPGUS and pKCaMV35S^2^GUS (replacing GUS), to design the plasmids pKMUASCsV8CP-KP4His and CaMV35S^2^-KP4His. The pKMUASCsV8CP construct was also designed to serve as a vector control (VC).

### Transient and Transgenic Assays of Promoter Clones

The native and chimeric promoters along with CaMV35S and CaMV35S^2^ (cloned in pUCPMAGUS) were electroporated into viable tobacco protoplasts following standard protocols (Acharya et al., 2014). Transient GUS activities from respective promoter constructs were calculated biochemically by fluorimetric GUS assay (Jefferson, 1987). For transient Agro-infiltration assays of the promoter constructs in tobacco, petunia, and tomato, *Agrobacterium tumefaciens* strain LBA4404 was transformed with respective promoter constructs following the freeze-thaw method as described previously (Chen et al., 1994). Leaves of whole plants of tobacco, petunia and tomato were mechanically infiltrated with individual *Agrobacterium* constructs (Yang et al., 2000). Quantitative fluorimetric GUS assay for each promoter construct was carried out 3–4 days’ post-infiltration (Jefferson, 1987; Ranjan et al., 2011; Acharya et al., 2014).

For comparative GUS activity analysis in agro-infiltrated leaves expressing CsVMV native deletion fragments cloned in pKYLXGUS, pKMUASCsV8CPGUS, pKMUAS35SCPGUS, pK35SGUS, and pK35S^2^GUS fragments; total leaf protein from transgenic seedlings (21 days old) were isolated and GUS activity analysis was performed according to standard protocols (Bradford, 1976; Jefferson, 1987). Histochemical GUS staining of 21 days old transgenic tobacco seedlings (T_2_ generation) was performed according to a previously published protocol (Jefferson et al., 1987).

### Production of Transgenic Tobacco Plants by Tobacco Leaf Disk Transformation

The native and chimeric promoter fragments along with *KP4-His* and VC constructs were used to transform tobacco plants by *Agrobacterium*-mediated gene transfer (Dey and Maiti, 1999) using LBA4404 strain. On an average, seventeen to eighteen T_0_ transgenic tobacco lines were generated for each of the constructs. All transgenic plants showed normal growth and development. Subsequently, the T_1_ generation seeds were subjected to segregation analysis (Patro et al., 2015). Transgenic lines with a chi-square value of <1.5 (*p* ≤ 0.05) were considered to be true transgenic lines. Independently re-generated transgenic lines showing proper segregation ratios (Kan^R^:Kan^S^::3:1) were selected and respective T_2_ generation transgenic plants were propagated in greenhouse conditions [photoperiod: 16/8 h (light/dark), light intensity: 220 μmol/m^2^/s, temperature: 28 ± 2°C and humidity: 70–75%]. The transgenic tobacco plants expressing *KP4-His* along with VC lines were also compared for their agronomical parameters such as plant height, leaf architecture, seed fertility and flowering time. Likewise, the GUS activities of the transgenic plants expressing pKYLXGUS, pKMUASCsV8CPGUS, pKMUAS35SCPGUS, pK35SGUS, and pK35S^2^GUS was performed following the standard protocol (Bradford, 1976; Jefferson et al., 1987).

### Gene Integration Assays

Total genomic DNA was extracted from 21-days old T_2_ transgenic seedlings of individual lines expressing VC and pKMUASCsV8CP-*KP4His*, respectively (Dellaporta et al., 1983). Gene integration assays for *uidA, KP4*, *rbcSE*9 and *npt*II were performed in respective transgenic lines using gene-specific primer sets (**Supplementary Table S1**). Transgenic lines expressing native and chimeric promoter constructs along with *KP4-His* and VC plants were subjected to PCR amplification (of the genes mentioned above) following the standard protocol using *Taq* DNA Polymerase (Thermo Fischer Scientific; Cat No. #EP0402) having proofreading activity (Kumar et al., 2011) and all the above amplicons were sequenced by Sanger sequencing method employing a sequencer (Applied Biosystems 3500 Genetic Analyzer).

### Southern Blotting

Briefly, 20 μg of genomic DNA isolated from selected T_1_ progenies of transgenic and VC lines was digested using the *Xho*I restriction enzyme (Fermentas ER0691) overnight (16 h) at 37°C, electrophoresed on 0.8% agarose gel, blotted on nylon membrane (nylon-N+, Amersham) and finally probed with PCR amplified αP^32^-labeled *GUS* and *KP4-His* probes using specific primers (**Supplementary Table S1**). The entire procedure of Southern Blotting was performed following a previously described protocol (Sambrook et al., 1989; Sarkar et al., 2015).

### Quantitative Real-Time PCR Analysis

Total RNA from transgenic tobacco seedlings expressing the respective constructs were isolated using the RNeasy plant mini kit (Qiagen, Chatsworth, United States) and cDNA synthesis was performed. After that, Real-time PCR analysis was performed using Premix Ex TaqTM II (Perfect Real Time, Takara Bio Inc., Japan) employing the Opticon-2Real-time PCR (MJ Research, Bio-Rad; Model; CFD-3220). The transcript levels for individual constructs were calculated by the 2^-ΔΔC_T_^ (CT-comparative threshold cycle) method (Livak and Schmittgen, 2001) and expressed as fold change in comparison to respective controls where *18S* was used as the housekeeping gene (Kumar et al., 2012).

### Western Blot Analysis of T_2_ Generation KP4 Transgenic Plants

The uppermost fully expanded leaves of 6-week-old greenhouse grown plants (T_2_ generation) of KP4L2#2, KP4L2#3, KP4L2#4, KP4L2#7, KP4L2#11, and VC were extracted and crushed in liquid nitrogen to make a fine powder. Next, the total soluble protein (TSP) was extracted from the crushed samples using an extraction buffer containing 1X PBS with 0.05% β-mercaptoethanol and plant protease inhibitor cocktail (Sigma- P9599-5mL), pH 7.0. An aliquot of 10 μg of total soluble protein obtained from transgenic lines was resolved on 15% SDS-PAGE for Western blot analysis. For detection of the recombinant proteins, primary antibody specific to the hexa- (6x) His-tag [(Mouse monoclonal His-probe (SC-57598) and Rabbit polyclonal His-probe (SC-803)] was used following the standard protocol (Sarkar et al., 2015).

### Purification of KP4 From Transgenic Plants

KP4 was purified from 21-days-old transgenic KP4-His seedling (T_2_) using Ni-NTA agarose (Qiagen) according to the manufacturer’s instructions with slight modifications as described earlier (Ma et al., 2005). Briefly, the seedlings of KP4L2#2 transgenic lines were homogenized (in 1X PBS, 1 mM phenylmethylsulfonyl fluoride and 1.5% PVP-40), filtered through layers of miracloth and centrifuged at 14,000 *g* for 20 min at 4°C. The supernatant obtained was dialyzed against 50 mM Tris-HCl (pH 7.0) buffer for 24 h at 4°C and adjusted to 40% ammonium sulfate saturation. After centrifugation at 30,000 *g* for 40 min, the enriched fraction obtained as pellet was solubilized, dialyzed (overnight) and filtered through a 0.45 μM membrane filter, and loaded on to a Ni-NTA agarose column. Finally, the column was washed with wash buffer (2 mM imidazole, 20 mM Na_2_HPO_4_, 100 mM NaCl). Ni-NTA agarose-bound KP4 was eluted with elution buffer (500 mM imidazole, 20 mM Na_2_HPO_4_, 250 mM NaCl). The eluted fractions were dialyzed extensively in 1x PBS and concentrated with a centrifugal filter device (Amicon 3 kDa cut-off).

### Purification of KP4 From Interstitial Fluid (IF) of Transgenic Plants

The extraction of IF from transgenic leaves (T_2_) expressing KP4 was performed following the infiltration- centrifugation technique (Chatterjee et al., 2017). The IF obtained post-extraction was enriched by 40% ammonium sulfate saturation and purified using Ni-NTA affinity purification. Finally, the eluted fractions were dialyzed and concentrated to detect KP4.

### *In Vitro* Agar-Based Killing Zone Assay

For agar-based killing zone assay, we followed a previously published protocol with slight modifications (Allen et al., 2011). The plant material for plate-diffusion assay was prepared by crushing 50 mg of leaf material in 1 ml of sterile 1X Phosphate buffered saline (PBS, pH 7.0). *A. alternata* (MTCC No.10833) and *P. exigua* var. *exigua* (MTCC No. 2321) cultures procured from MTCC (Microbial Type Culture Collection, Chandigarh, India) were grown on Potato Carrot Agar (PCA) medium (HiMedia- M696-500G) for 3 and 5 days, respectively. Fine wells were cut into the agar with the help of a cork borer (diameter = 0.5 cm) and 100 μl of the enriched test plant material prepared from both transgenic and VC lines were added to each well on either side of the fungal growth. Two other wells containing 100 μl each of sterile water and PBS were used as controls. The plates were then incubated at 25°C under constant light for *A. alternata* and, 12:12 h light:dark cycle for *P. exigua* var. *exigua.* The plates were photographed when clear zones of inhibition started to appear around the point of application of test samples.

To compare the protection efficacies of MUASCsV8CP and CaMV35S^2^, we cloned the *KP4-His* downstream of the above promoters to generate pKMUASCsV8CP-KP4His and pKCaMV35S^2^-KP4His constructs. The recombinant constructs were then agro-infiltrated in healthy tobacco leaves. After 3–4 days of incubation, the infiltrated leaf segments were excised and total protein was isolated as described above. Equal concentrations of protein from both the constructs were used for the agar-based killing zone assays against *A. alternata* and *P. exigua* var. *exigua.*

### *In Vivo* Disease Resistance Assays on Detached Leaves

Fungal resistance assays on detached leaves (employing *A. alternata* and *P. exigua* var. *exigua*) were performed according to a standard protocol with minor modifications (Emani et al., 2003). Briefly, young and healthy leaves were collected from 3 to 4 weeks old greenhouse grown plants and kept on wet paper towels in a petri-dish. On the other hand, *A. alternata* and *P. exigua* var. *exigua* were grown for 2 weeks on Potato Carrot Agar and Oat Meal Agar medium, respectively. Finally, an agar plug containing full mycelial growth was removed with the help of a cork borer (diameter = 0.5 cm) and agar plugs containing *A. alternata* and *P. exigua* var. *exigua* were placed on the adaxial surface of the leaves. In case of *P. exigua* var. *exigua* assay, the leaves were wounded with the help of a sterile needle at multiple points (6–7). Alongside, a mock leaf was kept to evaluate the effects of wounding inflicted by the needle. The petri-plates were sealed and kept in dark at 28°C. The leaves were photographed on the 14th and 18th day post-inoculation (dpi) of *A. alternata* and *P. exigua* var. *exigua*, respectively. The lesion area percentage was calculated with the help of millimeter graph paper method (Pandey and Singh, 2011).

### Whole Plant Assays Against *A. alternata* and *P. exigua* var. *exigua*

The whole plant assays were performed following a previously published protocol. Healthy leaves of 8-weeks-old transgenic KP4L2#2, KP4L2#3, and VC plants were sprayed with conidial suspension of *A.alternata* and *P. exigua* var. *exigua* (Bithell and Stewart, 1998) using a sprayer. In case of *P. exigua* var. *exigua*, however, the leaves were wounded before applying the conidial suspension. The greenhouse conditions [photoperiod: 12:12 h (light:dark), light intensity: 220 μmol/m^2^/s, temperature: 25 ± 2°C and humidity: 90–95%] were kept extremely humid and the symptoms were recorded/photographed 20 dpi. The lesion area percentage was recorded as described above.

### Statistical Analysis

All experiment procedures were carried out in at least three independent replicates and the statistical analyses was performed using one-way analysis of variance (ANOVA) where a *P*-value of <0.05 was considered as significant.

## Results

### Molecular Characterization of the Full-Length Transcript Promoter(-565 to +166) of CsVMV

**FIGURE 1:**
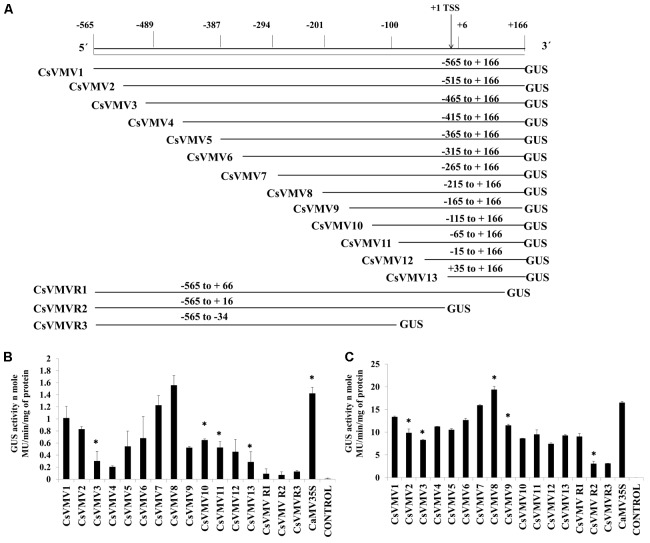
5′–3′ and 3′–5′ end deletion analysis of CsVMV promoter fragment. **(A)** Schematic representation of deletion constructs (16 numbers) coupled to *GUS* reporter gene and their respective co-ordinates. The relative positions of TATA-box and TSS are shown. **(B)** Transient protoplast GUS expression analysis of the above mentioned deletion constructs in tobacco protoplasts. **(C)** Transient GUS activities of the promoter deletion constructs in whole plant of *Nicotiana tabacum* Samsun NN following Agro-infiltration assays. Average GUS activities (nmole MU/min/mg protein) of three independent experiments obtained from transformed protoplasts and agro-infiltration assays for above deletion constructs were presented with respective SD. Asterisks indicate level of significance where “^∗∗^” is more significant than “^∗^”.

The transient activities of each of the sixteen 5′ and 3′-end deletion constructs coupled to GUS reporter (pUCPMAGUS-CsVMV1 to pUCPMAGUS-CsVMVR3) (**Figure 1A**) were evaluated in tobacco protoplasts (cv. *Xanthi Brad*). **Figure 1B** depicts the average GUS activities obtained for each of the deletion constructs along with the CaMV35S promoter and an empty vector (EV) control with respective standard deviations (SDs). The data obtained clearly suggests that the relative GUS activity of the CsVMV8 (-215 to +166) was almost equivalent (∼1.1 times) to that obtained for the CaMV35S promoter; while the CsVMV7 (-256 to +166) ranked second. Furthermore, the TATA-less fragments CsVMVR2 (-565 to +16) and CsVMVR3 (-565 to -34) showed diminished GUS activities.

Additionally, we evaluated the transient activities of the above constructs in whole plants of *Nicotiana tabacum* Samsun NN by agro-infiltration assays. The average GUS activities along with respective SD were presented in **Figure 1C**. The data obtained was in accordance with that of the protoplast assays.

**FIGURE 2:**
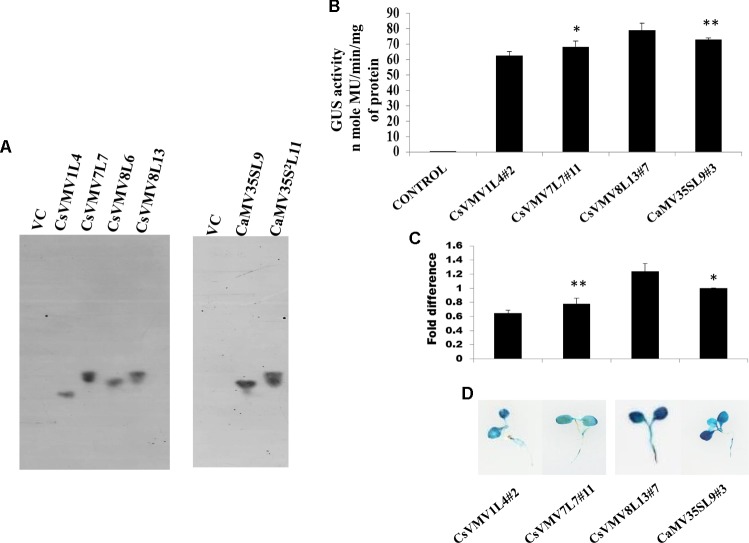
Transgenic activity analyses of CsVMV1L4, CsVMV7L7, CsVMV8L13, and CaMV35SL9. **(A)** Southern blot analyses to investigate the copy number of *uidA* gene in transgenic tobacco lines (T_1_ generation). **(B)** Stable transgenic GUS expression analyses of the above promoter constructs were presented with their corresponding SD. **(C)** Relative fold difference of *uidA* transcript levels in 21-day old transgenic seedlings under the control of respective promoter fragments. **(D)** Images of X-Gluc stained whole seedlings (T_2_ generation) expressing CsVMV1L4#2, CsVMV7L7#11, CsVMV8L13#7, and CaMV35SL9#3 promoters coupled to *GUS* reporter gene. Asterisks indicate level of significance where “^∗∗^” is more significant than “^∗^”.

Finally, we raised transgenic tobacco plants by Agrobacterium mediated leaf disk transformation protocol as described in Methods. We successfully raised 7–10 independent transgenic lines expressing *uidA* gene under the control of CsVMV1, CsVMV7, CsVMV8, and CaMV35S promoter fragments, respectively. Next, we performed segregation and Southern-blot analysis of T_1_ progeny transgenic plants. We observed, single copy integration of the transgene *uidA* in the transgenic lines (T_1_ generation) CsVMV1L4, CsVMV7L7, CsVMV8L6, CsVMV8L13, CaMV35SL9, and CaMV35S^2^L11. Finally, from the Southern-blot analysis, those transgenic plants having single copy insertion of the transgene (*uidA*) and exhibiting appropriate segregation ratio (3:1), namely CsVMV1L4, CsVMV7L7, CsVMV8L13, and CaMV35SL9 were chosen for further analyses (**Figure 2A**).

We evaluated the stable GUS activities of 3-week old transgenic tobacco seedlings (T_2_ generation). On analysis of the results, we observed that the CsVMV8 showed ∼1.1 times stronger activity than that of the CaMV35S promoter (**Figure 2B**). Additionally, we performed qRT-PCR analysis of the transgenic plants as described in section “Materials and Methods.” The relative accumulation levels of *uidA*-mRNA level in transgenic plants (T_2_ generation) harboring the respective promoter constructs (viz. CsVMV1L4#2, CsVMV7L7#11, CsVMV8L13#7, and CaMV35SL9#3) confirmed ∼1.1-fold more expression of *uidA* under the control of CsVMV8 promoter in comparison to the CaMV35S promoter (**Figure 2C**). Finally, we obtained a fairly strong intensity of *X*-Gluc staining of 21 days-old transgenic seedlings expressing the CsVMV8 promoter (**Figure 2D**), in comparison to that obtained under other constructs.

### Characterization of MUASCsV8 Chimeric Promoter

**FIGURE 3:**
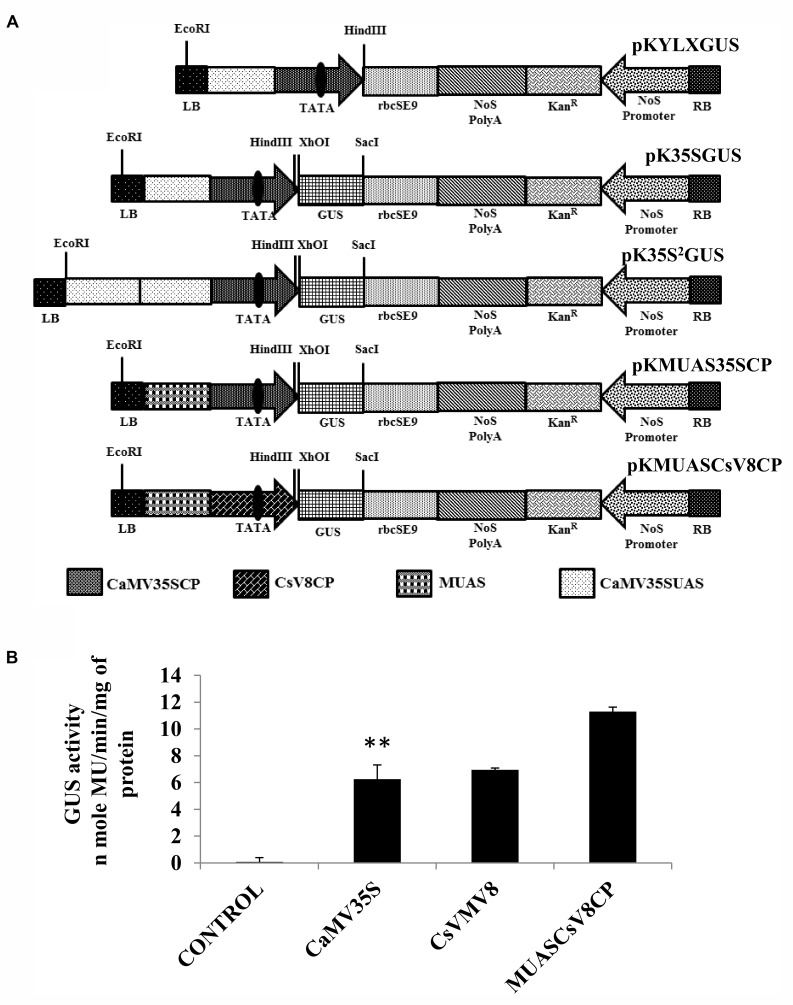
**(A)** Graphical representation of plant expression vectors harboring promoter constructs namely pKYLX (containing 35S promoter), pKYLXGUS, pK35S^2^GUS, pK35SGUS, pKMUAS35SCP, and pKMUASCsV8CP coupled to *GUS* reporter gene. The relative position of UAS, CP with TATA element and different essential components of the expression cassette were illustrated. **(B)** Transient activity analyses of the above mentioned recombinant promoters in tobacco protoplasts. Asterisks indicate level of significance where “^∗∗^” is more significant than “^∗^”.

**Figure 3A** represents the schematic map with essential components of the chimeric promoter MUASCsV8CP along with CaMV35S, CaMV35S^2^ and MUAS35SCP. Upon transient activity analysis in tobacco protoplasts (**Figure 3B**) and in whole plants of tobacco, petunia, and tomato (**Supplementary Figure S1**), we detected that the GUS activity of MUASCsV8CP was ∼2.4 times stronger as compared to the CaMV35S promoter.

**FIGURE 4:**
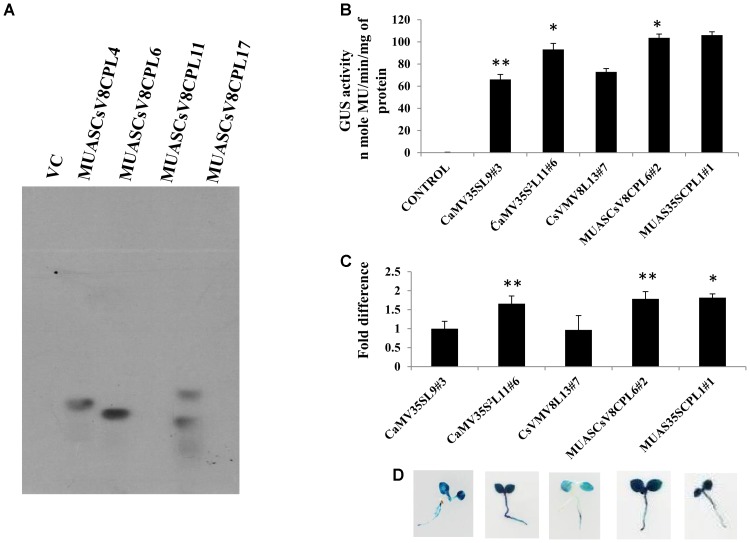
Transgenic activity analyses of CsVMV8L13, MUASCsV8CPL6, MUAS35SCPL1, CaMV35SL9, and CaMV35S^2^L11. **(A)** Southern blot analyses performed to investigate *uidA* gene copy number in transgenic tobacco lines. **(B)** GUS expression analyses of the above promoter constructs in 21-day old transgenic seedlings. **(C)** Each bar represents the relative fold difference of *uidA* transcript levels of the respective constructs. The mean relative fold differences obtained from three independent experiments with respective SD for each promoter construct was presented. **(D)** Images of *X*-Gluc treated whole seedlings (T_2_ generation) expressing GUS under the control of CsVMV8L13#7, MUASCsV8CPL6#2, MUAS35SCPL1#1, CaMV35SL9#3 and CaMV35S^2^L11#6 promoter constructs. Asterisks indicate level of significance where “^∗∗^” is more significant than “^∗^”.

We raised transgenic plants harboring the MUASCsV8CP-GUS and CaMV35S^2^-GUS (a modified version of CaMV35S with double enhancer domain) (Kay et al., 1987) constructs individually as already described. Southern blot analysis was performed to determine the copy number of the *uidA* transgene in above plant lines (T_1_ generation). The appearance of distinct southern-positive bands in MUASCsV8CPL4 and MUASCsV8CPL6 indicate integration of the transgene construct as a single copy insertion in the T_1_ progeny plant genome (**Figure 4A**). The progenies (T_2_ generation) of line MUASCsV8CPL6 exhibited expected segregation ratio and was chosen for further analyses.

We measured the GUS activities of the respective constructs using 21 days-old T_2_ transgenic seedlings having the CaMV35S, CaMV35S^2^, MUASCsV8CP, and MUAS35SCP promoters. The data obtained clearly showed that the chimeric promoter MUASCsV8CP, reported in this study has ∼2.4 and ∼1.1 times enhanced GUS activity as compared to CaMV35S and CaMV35S^2^ promoters, respectively. The data also indicates toward the equivalence in activities of MUASCsV8CP and MUAS35SCP promoters (**Figure 4B**).

Additionally, we performed the Real-time PCR analysis and histochemical *X*-Gluc staining of the transgenic seedlings expressing *GUS* under the control of CaMV35S, CaMV35S^2^, MUASCsV8CP, and MUAS35SCP promoters. The fold change as shown in **Figure 4C** indicates that the *uidA*-mRNA accumulation in MUASCsV8CP was nearly 1.8 times and 1.1 times as compared to CaMV35S and CaMV35S^2^ promoters, respectively, while MUASCsV8CP and MUAS35SCP showed the previous trend of being equivalent. Finally, the histochemical staining also suggested strongest intensity using MUASCsV8CP and MUAS35SCP chimeric promoters (**Figure 4D**).

### Generation of Tobacco Lines Expressing Totiviral Killer Protein (KP4) Under the Control of MUASCsV8CP Chimeric Promoter

**FIGURE 5:**
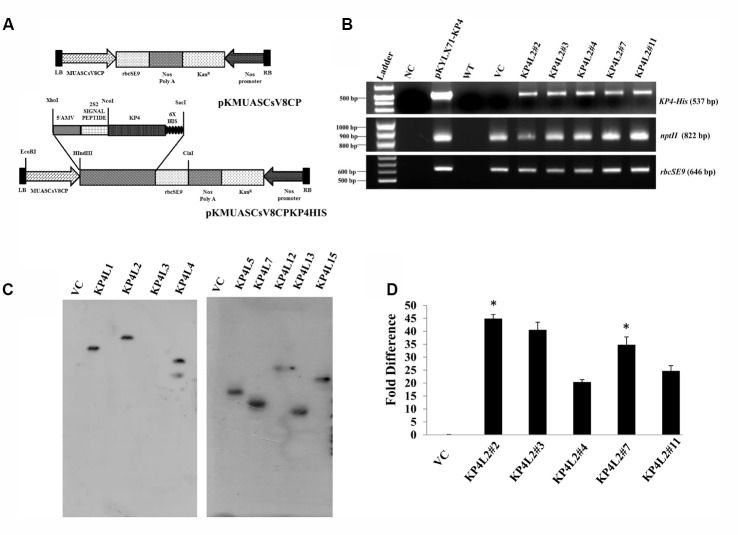
Gene-integration analyses of transgenic plants expressing KP4. **(A)** Schematic representation of the plant transformation vectors for the expression of KP4. **(B)** PCR amplification of different genes viz. (i) *KP4* (ii) *rbcSE9*, and (iii) *nptII* from transgenic plant lines KP4L2#2, KP4L2#3, KP4L2#4, KP4L2#7, and KP4L2#11. The *KP4* amplicon was electrophoresed along with a 1 kb DNA ladder (BioLit ProxiO 1 kb DNA Ladder plus BLL007) while the amplicons of *rbcSE9* and *nptII* were electrophoresed along with a 100 bp Ladder (Thermo Scientific GeneRuler, 100 bp DNA Ladder #SM0241). **(C)** Southern blot analyses to check the copy number of the *KP4* transgene in the above transgenic plant lines. **(D)** Real-time PCR analyses to check the abundance of *KP4* m-RNA in different transgenic lines. Asterisks indicate level of significance where “^∗∗^” is more significant than “^∗^”.

Using Agrobacterium-mediated gene transformation method we raised 18 independent T_1_ generation transgenic tobacco plants (Kan^R^) expressing totiviral *KP4* driven by MUASCsV8CP. **Figure 5A** represents the schematic map of both *KP4*-His and VC constructs. We performed segregation analysis of T_1_ generation transgenic plants as described in section “Materials and Methods.” Out of eighteen transgenic lines, the progenies (T_1_ generation) of eight independent lines showed proper segregation ratio (Kan^R^: Kan^S^::3:1) and found to be Kanamycin (300 mg/L) resistant on half strength Murashige Skoog (MS) medium (Zhu et al., 1999) (**Supplementary Table S2**). Seeds from these selected plants (T_1_ generation) showing proper segregation ratios were propagated to get homozygous T_2_ transgenic plants.

### Molecular Analysis of the Transgenic Plants Expressing MUASCsV8CP-*KP4His*

The integration of transgenes was confirmed by PCR amplification using primers specific to *KP4* (537 bp), *nptII* (822 bp), and *rbcSE9* (646 bp) (**Supplementary Table S1**). We confirmed the integration of different components of KP4 expression construct in T_2_ progenies of transgenic plant lines KP4L2#2, KP4L2#3, KP4L2#4, KP4L2#7, and KP4L2#11 whereas no amplification for *KP4* was observed in the VC plant line (**Figure 5B**). We analyzed the sequence integrity of the amplicons and did not detect any mutations in the nucleotide sequences of *KP4, nptII*, and *rbcSE9*.

Next, Southern-blot analysis was performed with gDNA isolated from different *KP4*-His transgenic lines and a VC plant (T_1_ generation progenies). Southern analysis confirmed the single copy insertion of *KP4*-His transgene construct in KP4L1, KP4L2, KP4L5, KP4L7, KP4L12, KP4L13, and KP4L15 transgenic tobacco plants (**Figure 5C**). KP4L4 however, showed two copies of the transgene. Further, the transcript analysis of *KP4* in KP4L2 transgenic lines (selected on the basis of segregation analysis) was performed by Real-time PCR. The result obtained from independent transgenic lines KP4L2#2, KP4L2#3, KP4L2#4, KP4L2#7, and KP4L2#11 clearly showed the accumulation of *KP4* in respective transgenic lines in the following order: KP4L2#2 > KP4L2#3 > KP4L2#7 > KP4L2#11 > KP4L2#4 as shown in **Figure 5D**.

### Isolation of Total Soluble Protein (TSP) and Western Blot Analysis of KP4-His

**FIGURE 6:**
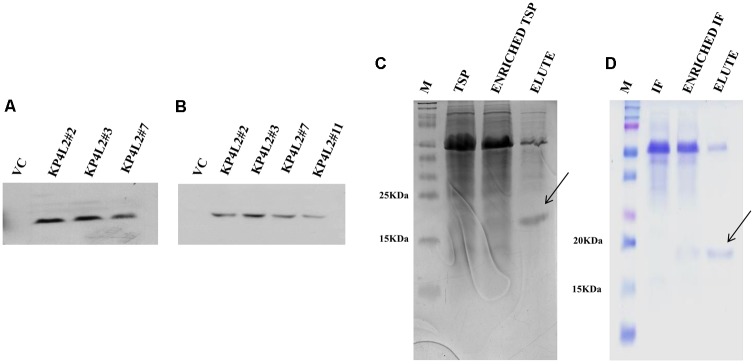
Western blot analysis of KP4 protein expression in transgenic tobacco plants using **(A)** anti-His polyclonal and **(B)** anti-His monoclonal primary antibody. Coomassie-stained 15% SDS-PAGE showing the partial purification of KP4 from **(C)** TSP and **(D)** IF fractions from pKMUASCsV8CP-KP4His transformed line KPL2#2.

In order to detect the KP4-His protein in transgenic plants, we isolated and concentrated TSP from individual transgenic seedlings (T_2_ generation). After isolation and quantification of TSP, we performed western blot analysis using anti-His polyclonal (**Figure 6A**) primary antibody as described in section “Materials and Methods.” The KP4-His expressed in transgenic tobacco plants representing 2S2aTP-KP4-His was seen as a prominent band, the molecular mass being ∼18 kDa along with a few faint non-specific bands. To avoid the appearance of any contaminating bands, we performed western blot with anti-His monoclonal primary antibody (**Figure 6B**). In this case, a discrete single band of ∼18 kDa indicating the presence of 2S2aTP-KP4-His in TSP was confirmed.

### Partial Purification of KP4 From Transgenic Tobacco Plants

To further detect the presence of KP4 in transgenic plants we performed nickel-nitrilotriacetic acid (Ni-NTA) affinity purification using TSP obtained from 21 days-old KP4L2#2 T_2_ transgenic plants as described in section “Materials and Methods.” The C-terminal 6xHis tag present in MUASCsV8CP-KP4-His (plant expression construct) facilitated the purification of recombinant KP4 using Ni-NTA resin. The SDS-PAGE analysis clearly showed a band of ∼18 kDa after 40% ammonium sulfate enrichment coupled to Ni-NTA affinity chromatography process (**Figure 6C**). We also observed a prominent contaminating band of ∼55 kDa which we assume might be of the RuBiSCO large subunit.

### Detection of KP4 from Interstitial Fluid (IF) of Transgenic Tobacco Plants

In order to confirm the accumulation of KP4 in the IF of transgenic plants, we isolated and partially purified the intercellular fluid (as described in section “Materials and Methods”) from the transgenic line KP4L2#2. The presence of 2S2aTP from *Arabidopsis* 2S2 protein gene facilitated the accumulation of KP4 in the IF. The 40% ammonium sulfate enrichment and Ni-NTA affinity purification module was adopted for purifying KP4 from the IF of transgenic plants. Appearance of distinct bands of ∼18 kDa in the eluted fraction (as shown in **Figure 6D**) indicated that the KP4 is properly targeted to the plant apoplast or intercellular space. In this case also we observed the band of ∼55 kDa as a contaminant.

### *In Vitro* Agar-Based Killing Zone Assay Against Foliar Fungal Pathogens

**FIGURE 7:**
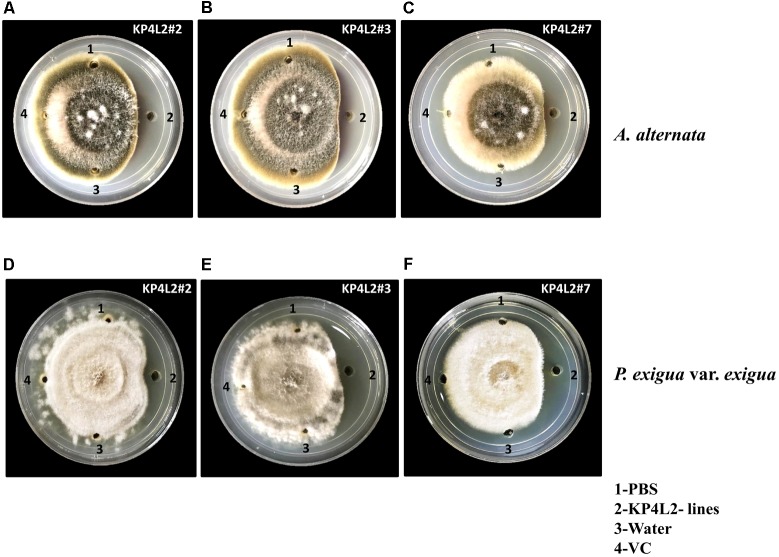
*In vitro* agar plate diffusion assays against **(A–C)** *Alternaria alternata* and **(D–F)** *Phoma exigua* var. *exigua*. Here, wells on each side of the fungal growth represent the VC, KP4L2#2, KP4L2#3, and KP4L2#7 plant materials grounded in 1X sterile PBS along with sterile water and buffer (PBS) taken as controls.

As KP4 blocks voltage-gated calcium channels of fungi, we sought to check the efficacy of plant-derived KP4 against two deuteromycetous fungal phyto-pathogens: *A. alternata* and *P. exigua* var. *exigua* using the ‘plate-diffusion’ assay (Allen et al., 2011). Enriched protein extracts for KP4L2#2, KP4L2#3, and KP4L2#7 were evaluated for anti-fungal activity as shown in **Figures 7A–C** (*A. alternata*) and **Figures 7D–F** (*P. exigua* var. *exigua*). It was evident that the KP4 activity represents a clear zone of inhibition around the point of application. This inhibition might be due to the blockage in calcium uptake by the fungal cells causing precocious hyphal disintegration.

### Comparative *in Vitro* Agar-Based Assays for MUASCsV8CP-KP4His and CaMV35S^2^-KP4His

Using KP4 as the gene of interest, we checked the efficacies of the MUASCsV8CP and CaMV35S^2^ promoters in the frame of fungal inhibition against *A. alternata* and *P. exigua* var. *exigua*. For this assay, we used transiently expressed KP4 from tobacco leaves infiltrated with pKMUASCsV8CP-KP4-His and pKCaMV35S^2^-KP4-His constructs (**Supplementary Figure S3**). Although we observed a slightly enhanced inhibition in case of pKMUASCsV8CP-KP4-His, we assume equivalence in activities of both promoter constructs.

### Disease Resistance Assays With *Alternaria alternata* and *Phoma exigua* var. *exigua*

Deuteromycetous necrotrophic fungal pathogen *Alternaria* and hemi-biotrophic pycnidial pathogen *Phoma* causes foliar diseases in a wide variety of vegetables and annual plants (Momol et al., 2004; Mamgain et al., 2013). *A. alternata* causes brown spot disease on tobacco which is characterized by the appearance of roughly circular necrotic spots with concentric rings surrounded by yellow halo. On the other-hand, *P. exigua* var. *exigua* causes Phoma blight or ragged leaf spot in tobacco which is characterized by the appearance of irregular brown to ashy gray spots. In order to evaluate the anti-fungal efficacy of transgenic plants expressing *KP4*, both *A. alternata* and *P. exigua* var. *exigua* were used as test pathogens. Prior to performing the bio-assays with the above fungal strains (procured from MTCC), we checked for their virulence toward infecting tobacco plants. We found that both the pathogens could successfully infect mature tobacco leaves and develop visible symptoms (data not shown).

**FIGURE 8:**
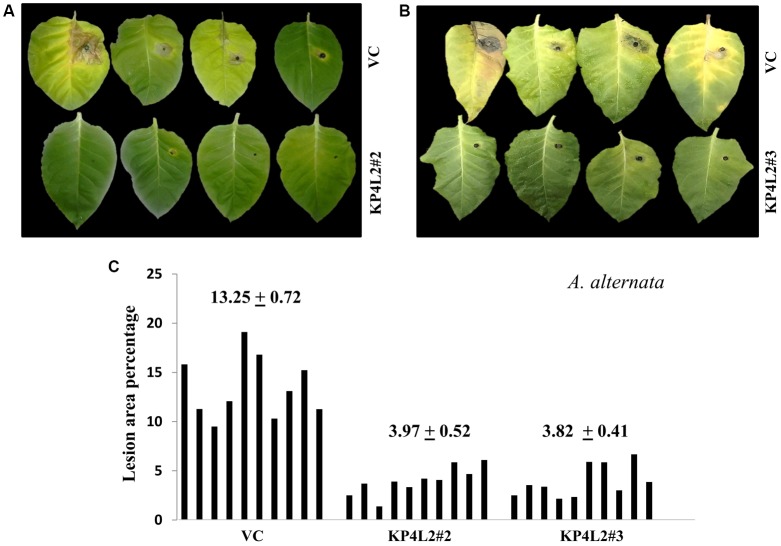
**(A,B)** *Alternaria alternata* disease resistance assays on detached leaves of VC, KP4L2#2 and KP4L2#3. An agar plug containing the full growth of the pathogen was placed on adaxial surface of the leaves. The images indicate lesion development 2 weeks post-infection. **(C)** Leaves from ten plant each of KP4L2#2, KP4L2#3, and VC were infected with one agar plug and subsequently lesion area percentage was measured by millimeter graph paper method. The numbers above each set of bars indicate the mean of percent lesion area ± standard error (SE) of the mean.

**FIGURE 9:**
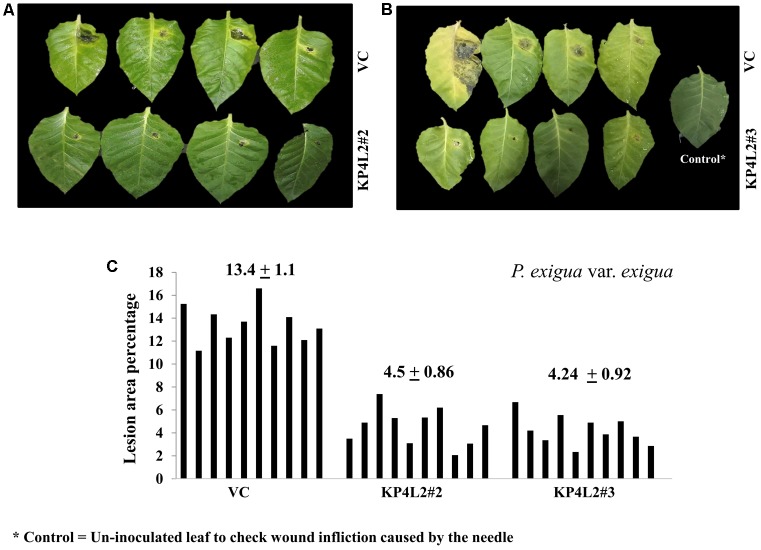
**(A,B)** *P. exigua* var. *exigua* disease resistance assays on detached leaves of VC, KP4L2#2, and KP4L2#3 indicating lesion development two and half weeks post-infection. **(C)** Graph showing lesion area percentage along with respective means and SE.

The disease resistance assays for both the pathogens were carried out with detached leaves kept on wet paper towels on petri dishes as described in section “Materials and Methods.” The detached-leaf technique is a routinely used method to determine the disease resistance of a particular transgenic plant against necrotrophic pathogens (Emani et al., 2003). In case of *A. alternata*, yellowish halos began to appear 7–8 days dpi while in *P. exigua* var. *exigua*, irregular brown spots appeared 10–11 dpi. The lesion area was measured 2 weeks post-inoculation for *A. alternata* (**Figures 8A,B**) while 18 dpi for *P. exigua* var. *exigua* (**Figures 9A,B**). The results revealed a high degree of protection in transgenic plants expressing *KP4* as compared to the VC, as supported by the observation that the leaves of transgenic plants were found to be unaffected or developed smaller lesions than the VC plants.

Following detached leaf assay, the lesion area percentage was determined using ‘Millimeter graph paper method’ as described in section “Materials and Methods.” We found, lesser lesion area in case of KP4 transgenic leaves as compared to the control plants (**Figures 8C**, **9C**). These results suggested an enhanced anti-fungal activity of KP4 against these foliar fungal pathogens, which can be considered as significant.

### Disease Resistance Assays Using Whole Plants Against *A. alternata* and *P. exigua* var. *exigua*

**FIGURE 10:**
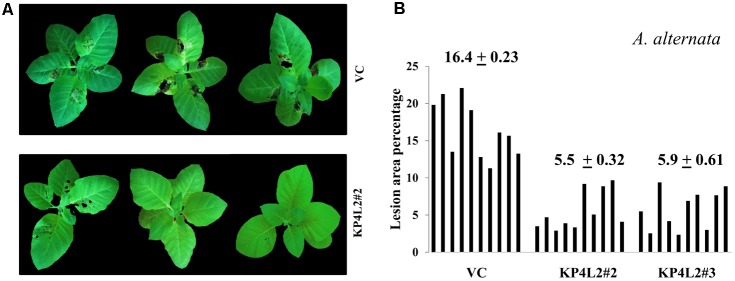
Whole plant assays against *A. alternata.* **(A)** Representative images of transgenic plant line KP4L2#2 along with VC 13 dpi. Ten plants each of KP4L#2, KP4L2#3, and VC were sprayed with conidial suspension of the fungus (10^7^ CFU/mL) to initiate infection. **(B)** The graph represents lesion area percentage showing symptoms ± SE calculated on the 13th dpi.

**FIGURE 11:**
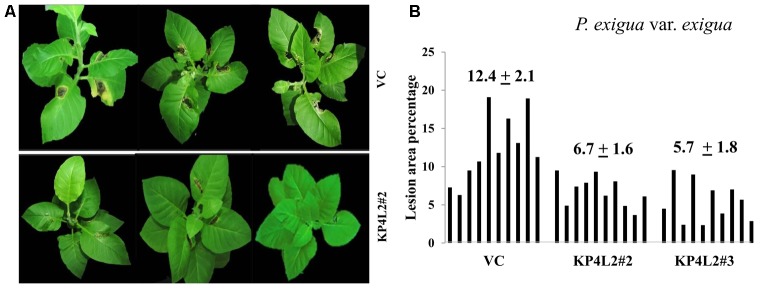
Whole plant assays against *P. exigua* var. *exigua*. **(A)** Representative images of transgenic plant line KP4L2#2 along with VC 20 dpi. Ten plants each of KP4L2#2, KP4L2#3, and VC were wounded with a sterile needle and conidial suspension of the fungus (10^7^ CFU/mL) was applied to initiate infection. **(B)** The graph represents percent lesion area ± SE calculated on the 20th dpi.

As described in section “Materials and Methods,” three independent experiments were carried out with whole plants of transgenic and VC lines. In all sets of experiments, the VC leaves developed distinct lesions much earlier than the *KP4* transgenic plants. Significant lesions of *A. alternata* infection were observed 9–10 dpi in VC plants (**Figure 10A**). On an average, we observed that the VC plants showed 16.4% lesion area, while KP4L2#2 and KP4L2#3 showed 5.5 and 5.9% lesion areas, respectively (**Figure 10B**). Likewise, we observed higher degree of lesions caused by *P. exigua* var. *exigua* in VC plants as compared to the KP4 transgenic lines KP4L2#2 and KP4L2#3 (**Figure 11A**). We observed 12.4% lesion area (as a mean value) in VC plants, in comparison to 6.7 and 5.7% lesion areas in KP4L2#2 and KP4L2#3 transgenic lines, respectively (**Figure 11B**).

Consistent with our results of detached-leaf assays for *A. alternata* and *P. exigua* var. *exigua*, we found significantly lesser leaf-area exhibiting symptoms in case of *KP4* transgenic leaves as compared to the VC plants. Although, some of the leaves showed inconsistent results, the percent lesion area clearly suggested inhibition and/or delayed lesion development in transgenic plants expressing *KP4*. The inhibition found in detached-leaf and whole plant assays further proved the anti-fungal efficacy of KP4 against both *A. alternata* and *P. exigua* var. *exigua*.

## Discussion

The emergence of various fungicide-resistant fungal-isolates deeply necessitates the evaluation of novel candidate genes coding for anti-microbial compounds. In the current scenario, where great genetic diversity and resistant isolates of pathogens thrive in nature, production of resistant crop varieties by transgenic approach is considered one of the easiest and effective device to control recalcitrant plant pathogens (Mundt, 2014). There are recent reports where in expression of transgenes in plants has conferred increased resistance against fungal pathogens, such as transgenic plants engineered with *glucanase* gene from alfalfa (Singh et al., 2014); *ERF1-V* gene from *Haynaldia villosa* (Xing et al., 2017) and *PSS1* from *Arabidopsis thaliana* (Wang et al., 2017). Likewise, this study is a nascent report of significant anti-fungal activity exerted by recombinant KP4 on two deuteromycetous foliar pathogenic fungi *A. alternata* and *Phoma exigua* var. *exigua*.

Targeted gene expression in a transgenic platform lies under the control of the key genetic regulator ‘promoter’ which determines its spatio-temporal regulation. Designing of synthetic promoters can be of immense importance in plant modification through genetic engineering that can withstand biotic stress while also ensuring maximum productivity in the field of translational biology (Dey et al., 2015). In this context, we have developed a near constitutive synthetic promoter “MUASCsV8CP” having altered *cis*-architecture (as described in section “Materials and Methods”) with higher activity. It showed different levels of constitutive expression in different plant parts as follows: leaf > stem > root (**Supplementary Figure S2**). Owing to the above features, it may appear as an important tool for plant modification. It can be efficiently used in combination with other caulimoviral promoters like CaMV35S and/or CaMV35S^2^ for co-expressing multiple genes (gene pyramiding) in a plant cell to avoid unwanted genetic recombination. Furthermore, the MUASCsV8CP can stand as a substitute for CaMV35S^2^ promoter in plant biotechnology-based applications as its activity is nearly comparable to that of the latter. Our *in vitro* agar-based well diffusion assays revealed that the recombinant KP4 expressed under both CaMV35S^2^ and MUASCsV8CP promoters showed almost equal zone of inhibitions against *A. alternata* and *P. exigua* var. *exigua*, suggesting that the newly designed promoter can also be capable of imparting protection against phytopathogens.

Keeping in mind the above facts, we attempted to express a gene displaying anti-fungal properties under the control of our newly designed chimeric promoter MUASCsV8CP and evaluated its ability to inhibit the growth of phyto-pathogenic fungi. Here, *KP4* was chosen as the gene of interest based upon the inter-strain hindrance found in double-stranded DNA totiviruses. Having structural similarity to the scorpion toxin AaHII, it was proposed and eventually proved that KP4 prevents uptake of calcium by fungal cells. As a result, it prevents hyphal tip growth, budding and viability of the fungi. KP4 has no sequence similarity to any other member of the ‘killer protein’ gene family and is extremely basic in nature with a pI of 9.0. The KP4 is known to be composed of five-stranded antiparallel β-sheet and two antiparallel α-helices that are stabilized by five disulfide bonds. It has been reported that KP4 has no apparent effect on the viability or subcellular structures of human, plant or insect cell lines as well as found to be harmless to soil inhabitants. Moreover, KP4 degrades in the stomach fluid and has no sequence resemblance with any known allergens (Schlaich et al., 2006; Allen et al., 2011).

A total of eight transgenic plant lines harboring MUASCsV8CP-KP4His (KP4L1, KP4L2, KP4L5, KP4L7, KP4L12, KP4L13, KP4L15, and KP4L17) showed normal agronomical parameters with proper segregation ratio but slight delay in root growth (**Supplementary Table S2**). However, this delay was not as evident as observed by Allen et al. (2008) probably because purified KP4 was applied exogenously in their experiments. The distinct southern positive bands in KP4L1, KP4L2, KP4L5, KP4L7, KP4L12, KP4L13, and KP4L15 (T_1_ progenies) indicated the single copy insertion of the transgene construct. We chose KP4L2 for our further studies as it exhibited good growth and maximum number of homozygous lines. Molecular analysis of the transgenic plants expressing KP4 confirmed the integration of *KP4* in T_2_ progenies of KP4L2 lines viz. KP4L2#2, KP4L2#3, KP4L2#4, KP4L2#7, and KP4L2#11. Subsequently, Real-time PCR analysis of the above transgenic plants indicated toward the maximum transcript accumulation in KP4L2#2.

Plants monitor the apoplastic fluid, secrete hydrolytic enzymes and anti-microbial metabolites in response to fungal colonization which re-models the apoplast to become the first battleground for any plant-fungal interaction (Du et al., 2016; Ma et al., 2017). Hence, we targeted the chimeric construct harboring *KP4* toward the plant apoplast using an ‘apoplast targeting sequence’ from *Arabidopsis* 2S2 protein gene (Sarkar et al., 2015). Also, our chimeric construct had a 6X-His tag at the 3′-end which aided the detection and purification of the same. We successfully purified recombinant KP4 from both TSP and IF of KP4L2#2 via Ni-NTA affinity purification. The SDS-PAGE analysis clearly showed the presence of 18 kDa band which appeared to be unglycosylated (**Figures 6B,C**).

We found strong antifungal activity of the KP4 extract against the virulent phytopathogens namely *A. alternata* and *P. exigua* var. *exigua* in the *in vitro* agar plate diffusion assays. This report is in accordance with the earlier published results of Spelbrink et al. (2004) where KP4 was extrinsically applied and found to be active against *Fusarium graminearum*, the causal agent of devastating head blight of small grain cereals (Yang et al., 2013). Interestingly, KP4 was found to inhibit the growth and progression of *Phoma* which has been previously described to form pseudosclerotia that perennate in soil during unfavorable environmental conditions.

The ‘detached-leaf’ assay is one of the most extensively used approaches for scoring disease symptoms. There are previous reports where this assay has been successfully employed to determine the anti-fungal potency of various compounds against *Sclerotinia sclerotiorum, Cercospora arachidicola*, and *Blumeria graminis* (Chenault et al., 2005; Roy-Barman et al., 2006; Anuradha et al., 2008). Likewise, we scored the disease symptoms against *A. alternata* and *P. exigua* var. *exigua* using this technique and observed that the transgenic lines expressing *KP4* displayed substantial resistance against these foliar pathogens. The whole plant assays performed against *A. alternata* and *P. exigua* var. *exigua* also suggested effective anti-fungal protection offered by KP4 *in planta*.

Furthermore, we sought to investigate the anti-fungal activity of KP4 against *Verticillium dahliae* (MTCC No. 9998), which causes vascular wilt in a wide range of plant species (Fradin and Thomma, 2006). Interestingly, we did not observe any contemplating inhibition against this pathogen (**Supplementary Figure S4**) which may be due to its ability to form long-lasting resistant melanised clumps called microsclerotia in soil/media (Goud et al., 2003; Tian et al., 2016). Regardless, to our knowledge this is the first report of significant anti-fungal activity exhibited by transgenic plants expressing *KP4* against two deuteromycetous phytopathogens.

## Conclusion

Our study has a tripartite vision: firstly, the characterization of CsVMV promoter where CsVMV8 (-215 to +66) was found to be the highest expressing fragment; secondly, hybridization of the CsVMV8 fragment with UAS of MMV (-297 to -38) to develop an inter-molecularly shuffled recombinant promoter “MUASCsV8CP” and finally, expression of the totiviral KP4 under this chimeric promoter to develop transgenic tobacco resistant against two foliar pathogenic fungi – *A. alternata* and *P. exigua* var. *exigua*. This study provides insights into the development of potential candidates having in-built biotic-stress tolerance coupled to efficient caulimoviral promoters with discrete *cis*-organization in a transgenic approach for plant biotechnology.

## Author Contributions

IM and ND conceptualized the experiments. DD, AS, and ND designed the experiments and critically analyzed the results and wrote the paper. DD performed all the experiments.

## Conflict of Interest Statement

The authors declare that the research was conducted in the absence of any commercial or financial relationships that could be construed as a potential conflict of interest.

## Supplementary Material

FIGURE S1 Transient agro-infiltration assays to evaluate the GUS expression in whole plants of **(A)** *Nicotiana tabacum* **(B)** *Petunia hybrida*, and **(C)** *Solanum lycopersicum* expressing *GUS* under respective promoter constructs along with VC leaves.

FIGURE S2 Spatial distributions of GUS activities under VC, CaMV35S, CaMV35S^2^, and MUASCsV8CP promoters. GUS activities (in nmole MU/min/mg protein) from the **(A)** root, **(B)** stem, and **(C)** leaf of transgenic tobacco seedlings (21 days old) expressing GUS under the control of the above promoter constructs were measured. Average GUS activities from three independent experiments for each of the promoter construct were presented with corresponding SDs.

FIGURE S3 *In vitro* agar plate diffusion assays using transiently expressed KP4-His. Equal amounts of TSP extracted from tobacco leaves infiltrated with constructs MUASCsv8CP-KP4His and CaMV35S^2^-KP4His were used to compare the inhibitions conferred against **(A,B)** *Alternaria alternata* and **(C,D)** *Phoma exigua* var. *exigua*, respectively. Sterile PBS and water were used as controls.

FIGURE S4 *In vitro* agar plate diffusion assays against *Verticillium dahliae.* Wells on each side of the fungal growth represent the VC and KP4L2 lines viz. KP4L2#2 **(A)** and KP4L2#3 **(B)** plant materials grounded in 1X sterile PBS.

TABLE S1 List of oligonucleotides (primers) used in this study. ^∗^ The annealing temperature for all the above PCR/Real Time PCR primer sets was 58°C.

TABLE S2 Segregation analysis of T_1_ generation plants expressing *KP4-His.*
