# Supplementary figures and images for: Recombinant Promoter (MUASCsV8CP) Driven Totiviral Killer Protein 4 (KP4) Imparts Resistance Against Fungal Pathogens in Transgenic Tobacco
Source: Front Plant Sci. 2018 Mar 5;9:278. doi: 10.3389/fpls.2018.00278 (PMC5844984; doi:10.3389/fpls.2018.00278)

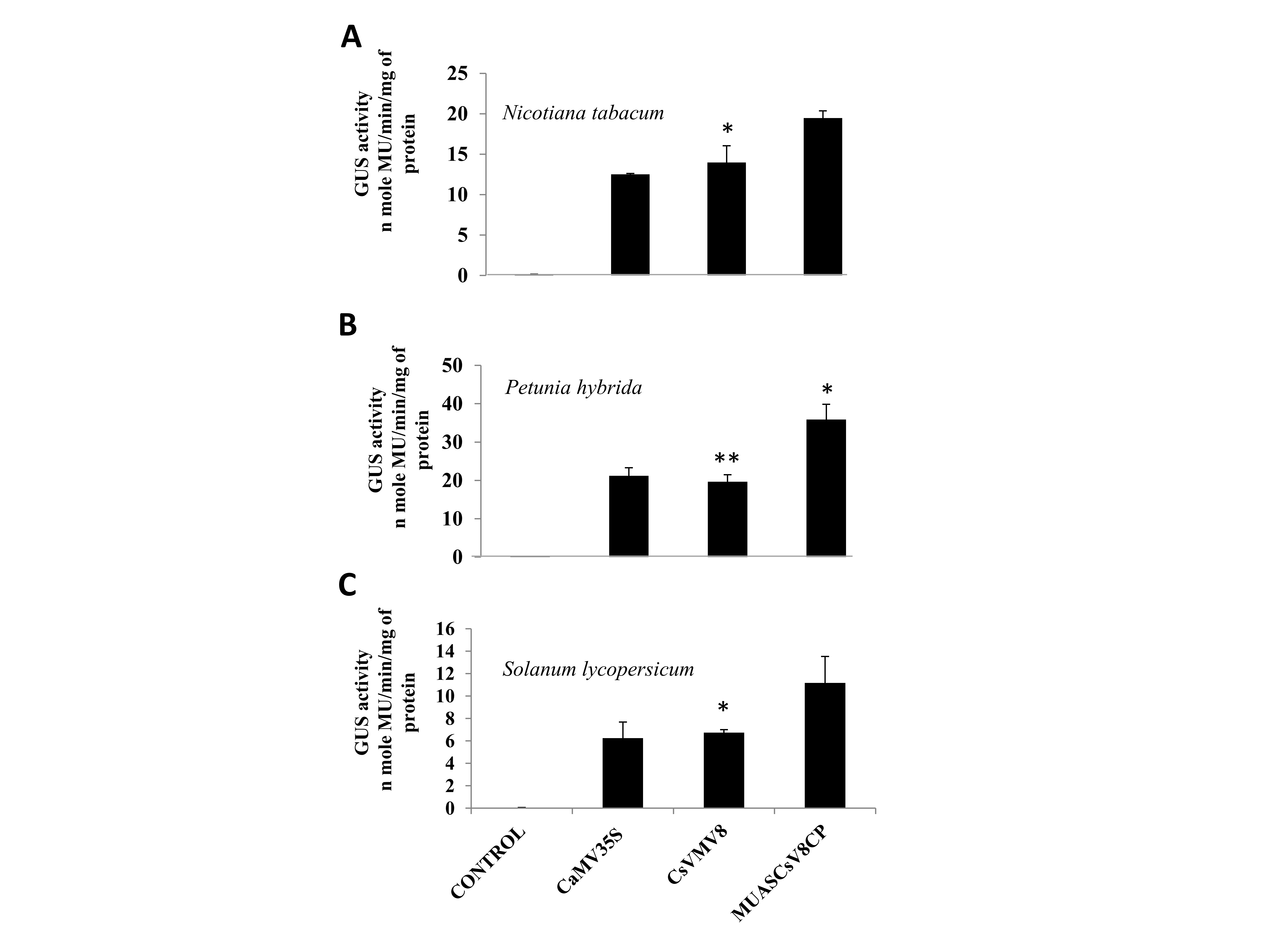

Supplement: FIGURE S1 — Transient agro-infiltration assays to evaluate the GUS expression in whole plants of (A) Nicotiana tabacum (B) Petunia hybrida, and (C) Solanum lycopersicum expressing GUS under respective promoter constructs along with VC leaves. [file Image_1.TIF]

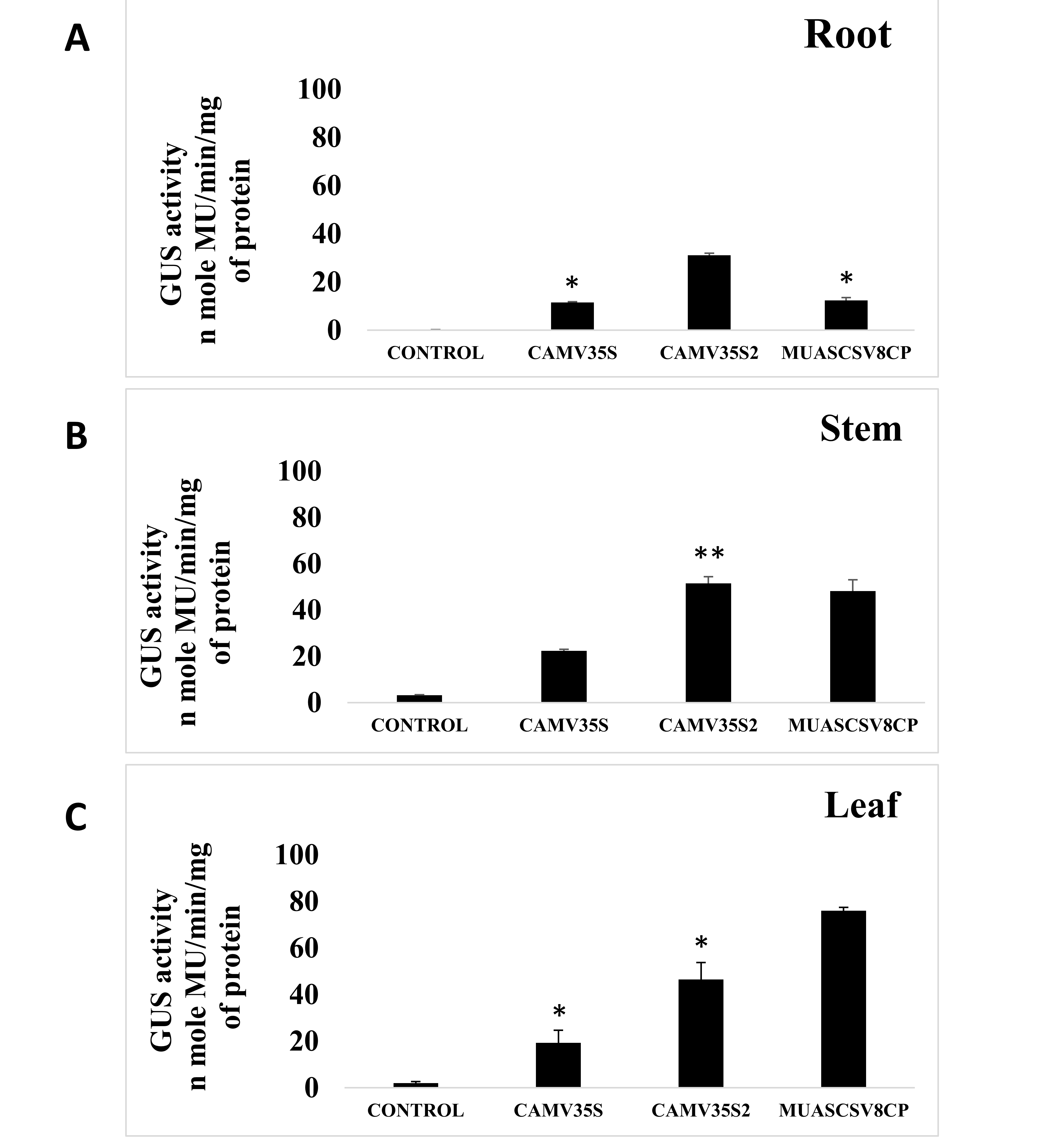

Supplement: FIGURE S2 — Spatial distributions of GUS activities under VC, CaMV35S, CaMV35S2, and MUASCsV8CP promoters. GUS activities (in nmole MU/min/mg protein) from the (A) root, (B) stem, and (C) leaf of transgenic tobacco seedlings (21 days old) expressing GUS under the control of the above promoter constructs were measured. Average GUS activities from three independent experiments for each of the promoter construct were presented with corresponding SDs. [file Image_2.TIF]

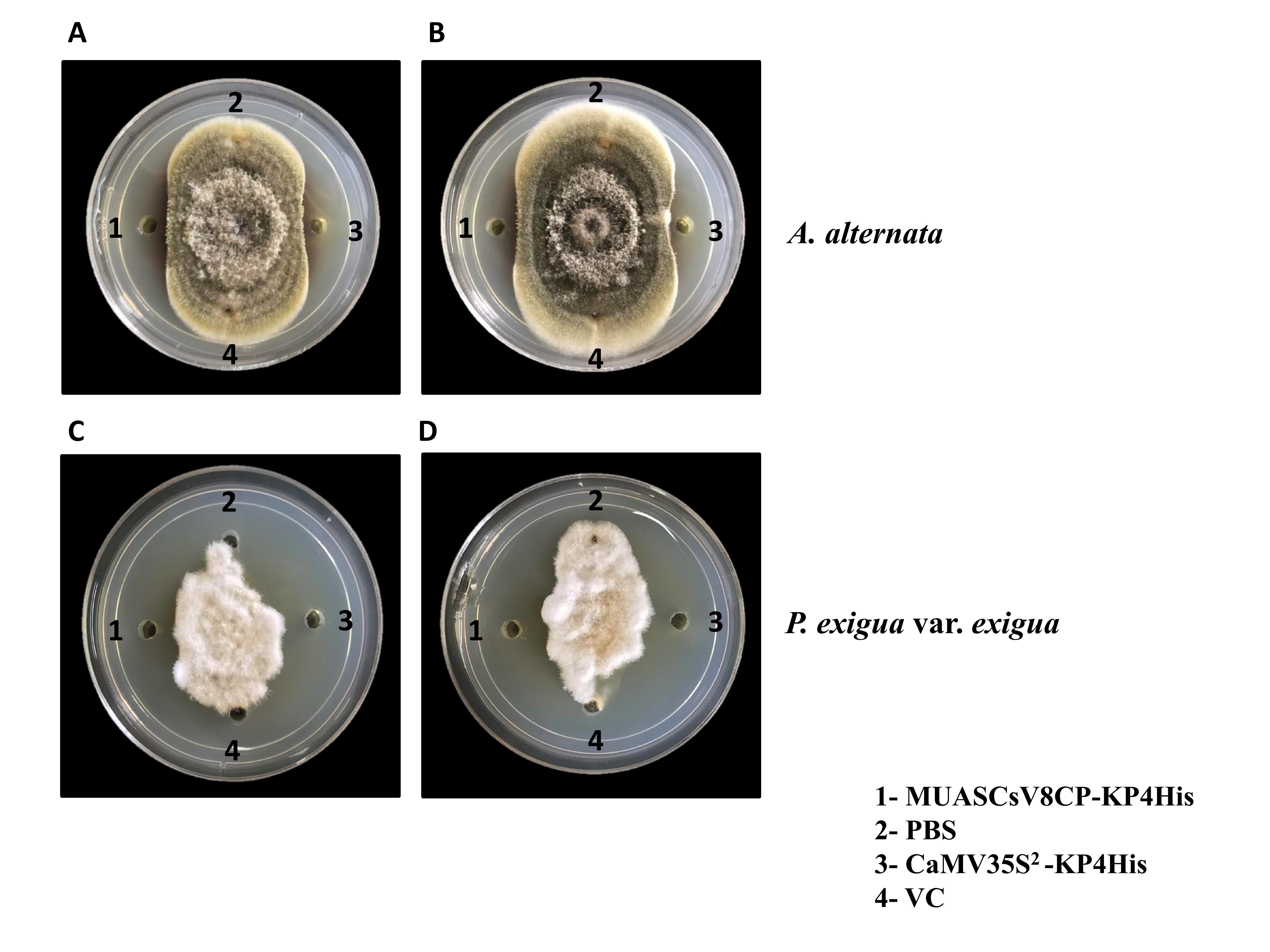

Supplement: FIGURE S3 — In vitro agar plate diffusion assays using transiently expressed KP4-His. Equal amounts of TSP extracted from tobacco leaves infiltrated with constructs MUASCsv8CP-KP4His and CaMV35S2-KP4His were used to compare the inhibitions conferred against (A,B) Alternaria alternata and (C,D) Phoma exigua var. exigua, respectively. Sterile PBS and water were used as controls. [file Image_3.TIF]

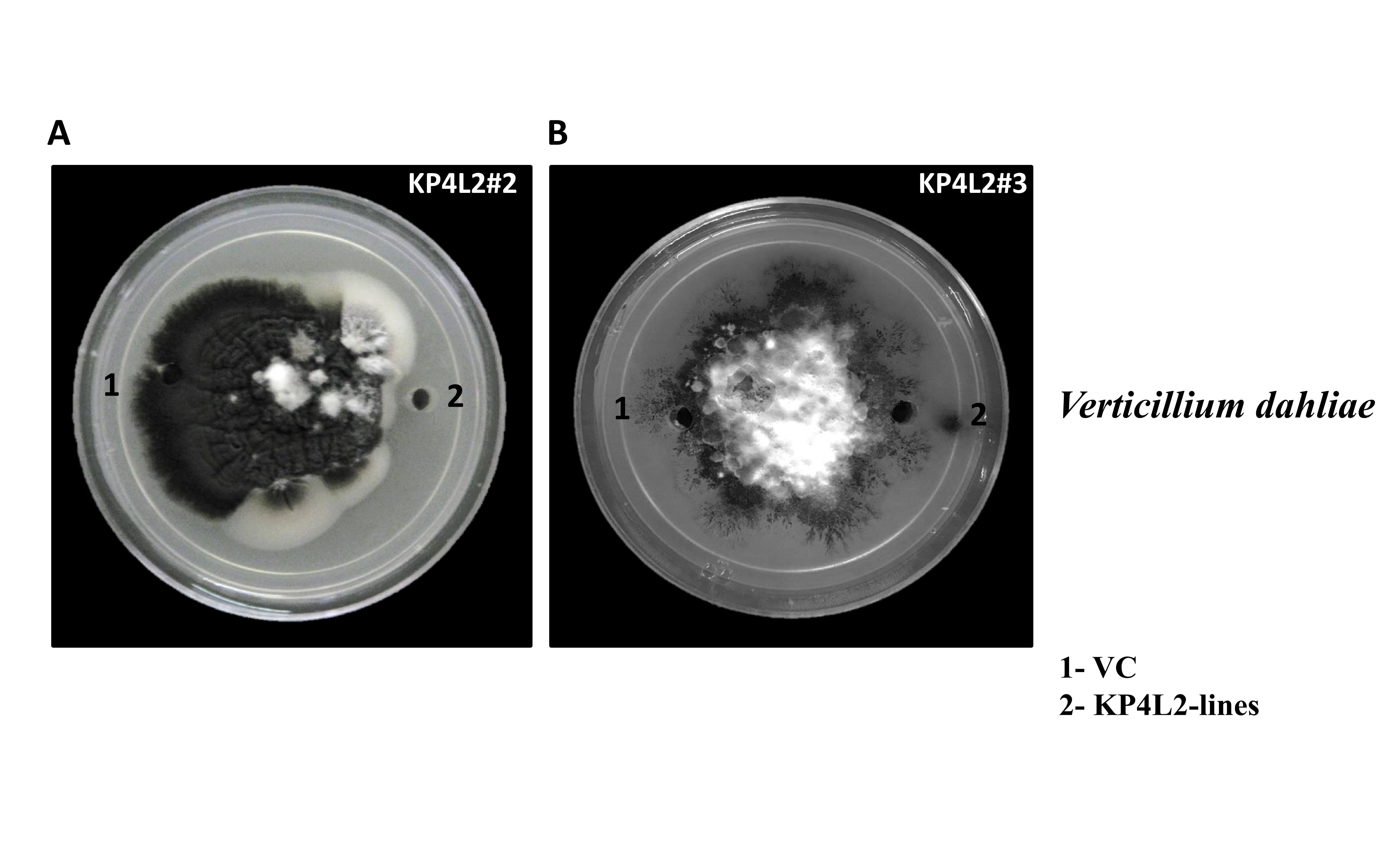

Supplement: FIGURE S4 — In vitro agar plate diffusion assays against Verticillium dahliae. Wells on each side of the fungal growth represent the VC and KP4L2 lines viz. KP4L2#2 (A) and KP4L2#3 (B) plant materials grounded in 1X sterile PBS. [file Image_4.TIF]
