# Supplementary material for: Recombinant Promoter (MUASCsV8CP) Driven Totiviral Killer Protein 4 (KP4) Imparts Resistance Against Fungal Pathogens in Transgenic Tobacco
Source: Front Plant Sci. 2018 Mar 5;9:278. doi: 10.3389/fpls.2018.00278 (PMC5844984; doi:10.3389/fpls.2018.00278)
Supplement: TABLE S1 — List of oligonucleotides (primers) used in this study. ∗ The annealing temperature for all the above PCR/Real Time PCR primer sets was 58°C. [file Table_1.docx]

| CONSTRUCT  **Supplementary Table 1:** List of oligonucleotides (primers) used in this study. | FORWARD PRIMER (5′ to 3′) | REVERSE PRIMER (5′ to 3′) |
| --- | --- | --- |
| CsVMV1 | GCGGGCGAATTCTGAATGGAAACGTATGGTTGT | ATGCAGAAGCTTCATAATTATTTCATATTAATT |
| CsVMV2 | GCGGGCGAATTCTCAAATCACAACAAAGCAGAA | ATGCAGAAGCTTCATAATTATTTCATATTAATT |
| CsVMV3 | GCGGGCGAATTCTTGGTCAAAAAAATTATCCAT | ATGCAGAAGCTTCATAATTATTTCATATTAATT |
| CsVMV4 | GCGGGCGAATTCTCAAGAATCCAATGTTTACGG | ATGCAGAAGCTTCATAATTATTTCATATTAATT |
| CsVMV5 | GCGGGCGAATTCAGCAAGAAGCAGATCAATATG | ATGCAGAAGCTTCATAATTATTTCATATTAATT |
| CsVMV6 | GCGGGCGAATTCGAAGAATGTACAGATACAAGA | ATGCAGAAGCTTCATAATTATTTCATATTAATT |
| CsVMV7 | GCGGGCGAATTCACGTAGAAATTGAAAAAGAAG | ATGCAGAAGCTTCATAATTATTTCATATTAATT |
| CsVMV8 | GCGGGCGAATTCGTAAGCACTGACGACAACAAT | ATGCAGAAGCTTCATAATTATTTCATATTAATT |
| CsVMV9 | GCGGGCGAATTCGAAAGAGACATAGAGGACACA | ATGCAGAAGCTTCATAATTATTTCATATTAATT |
| CsVMV10 | GCGGGCGAATTCTAACCTTATCACAAAGGAATC | ATGCAGAAGCTTCATAATTATTTCATATTAATT |
| CsVMV11 | GCGGGCGAATTCTTTCCGTGTCATTTTTGCCCT | ATGCAGAAGCTTCATAATTATTTCATATTAATT |
| CsVMV12 | GCGGGCGAATTCGGCATTTGTGAAAACAAGAAA | ATGCAGAAGCTTCATAATTATTTCATATTAATT |
| CsVMV13 | GCGGGCGAATTCTACTGAGGATACAACTTCAGA | ATGCAGAAGCTTCATAATTATTTCATATTAATT |
| CsVMVR1 | GCGGGCGAATTCTGAATGGAAACGTATGGTTGT | ATGCAGAAGCTTATTAAAACAAACATAAACA |
| CsVMVR2 | GCGGGCGAATTCTGAATGGAAACGTATGGTTGT | ATGCAGAAGCTTTACAAATTTCTCTGAAGTT |
| CsVMVR3 | GCGGGCGAATTCTGAATGGAAACGTATGGTTGT | ATGCAGAAGCTTCACCAAATTTTTTCTT |
| MUASCsV8CP | CCCGAATTCGTCGACTTCGTCCACAGACAT | ATGCAGAAGCTTCATAATTATTTCATATTAATT |
| MUAS35SCP | CCCGAATTCGTCGACTTCGTCCACAGACATCAA | GGGAAGCTTCCCGGGTCCTCTCCAAATGAAATGAAC |
| *GUS* (real time) | GATCGCGAAAACTGTGGAAT | TAATGAGTGACCGCATCGAA |
| *18S* (real time) | GCAAATTACCCAATCCTGAC | CTATTGGAGCTGGAATTACC |
| *KP4-HIS* | ATCGCTCGAGCGGATCTTTTTATTTTTA | ATGCAGGAGCTCTTAGTGATGGTGATGGTGATGACAAGAGTTAACATAGTTAAC |
| *KP4* (real time) | ACTGGATCCTATGGTTTGTGGTACTGGAAAC | ATCGCTCGAGACAAGAGTTAACATAGTT |
| *nptII* | ATGGCAATTACCTTATCCGCAACT | TCAGAAGAACTCGTCAAGAAGGCG |
| GUS (probe sequence for Southern blotting) | GATCGCGAAAACTGTGGAAT | TAATGAGTGACCGCATCGAA |
| *rbcSE9* | GCGTCCGGATCCGCTTTCGTTCGTATCATCGGTTTC | ATGTAGTCTAGATGATGCATGTTGTCAATCAATTGG |

* The annealing temperature for all the above PCR/Real Time PCR primer sets was 58°C.
