# Supplementary material for: Recombinant Promoter (MUASCsV8CP) Driven Totiviral Killer Protein 4 (KP4) Imparts Resistance Against Fungal Pathogens in Transgenic Tobacco
Source: Front Plant Sci. 2018 Mar 5;9:278. doi: 10.3389/fpls.2018.00278 (PMC5844984; doi:10.3389/fpls.2018.00278)
Supplement: TABLE S2 — Segregation analysis of T1 generation plants expressing KP4-His. [file Table_2.docx]

| INDEPENDENT LINE  **Supplementary Table 2:** Segregation analysis of T_1_ generation plants expressing *KP4-His.* | NUMBER OF SEEDS PLATED | COMMENTS/ SEEDLING GROWTH AND MORPHOLOGY | SELECTED LINES FOR ANALYSIS | *Chi*-square value | *P* value  (α = 0.05) |
| --- | --- | --- | --- | --- | --- |
| KP4L1 | 45 seeds  34:11 | Moderate to good growth, slight inhibition of root growth. | KP4L4#18, KP4L5#14. | 0.007 | 0.93 |
| KP4L2 | 43 seeds  33:10 | Good growth, slight inhibition of root growth. | KP4L2#2, KP4L2#3, KP4L2#4, KP4L2#7, KP4L2#11, KP4L2#20, KP4L2#28 | 0.07 | 0.79 |
| KP4L3 | 42 SEEDS  35:3 | Moderate growth |  | 5.92 | 0.01 |
| KP4L4 | 29 SEEDS  29:0 | Moderate to good growth |  | 9.66 | 0.001 |
| KP4L5 | 36 seeds  26:10 | Moderate growth, slight inhibition of root growth. | KP4L5#1, KP4L5#25. | 0.14 | 0.7 |
| KP4L 6 | 31 seeds  25:5 | Poor growth  Small roots |  | 1.11 | 0.29 |
| KP4L 7 | 38 seeds  28:10 | Moderate to good growth, slight inhibition of root growth. | KP4L7#2, KP4L7#5, KP4L7#9 | 0.03 | 0.85 |
| KP4L 8 | 45 seeds  28:17 | Moderate growth |  | 3.91 | 0.04 |
| KP4L 9 | 42 seeds  35:3 | Very poor growth, no roots |  | 5.74 | 0.01 |
| KP4L10 | 57 Seeds  52:2 | Moderate to poor growth, diminished root growth. |  | 12.53 | 0.0004 |
| KP4L11 | 47 seeds  all kanamycin sensitive |  |  | 141 | 1.6 |
| KP4L12 | 56 seeds  42:14 | Moderate growth, slight inhibition of root growth. | KP4L12#4, KP4L12#7, KP4L12#23 | 0 | 1.0 |
| KP4L13 | 45 seeds  32:13 | Good growth , slight inhibition of root growth | KP4L13#26 | 0.36 | 0.54 |
| KP4L14 | 40 seeds  21:19 | Poor growth, poor root growth |  | 10.8 | 0.001 |
| KP4L15 | 33 seeds  24:9 | Moderate growth | KP4L17#6, KP4L17#7. | 0.09 | 0.76 |
| KP4L16 | 48 seeds  33:12 | Moderate growth, moderate inhibition of root growth |  | 0.25 | 0.61 |
| KP4L17 | 39 seeds  28:9 | Good growth, slight inhibition of root growth. | KP4L17#4, KP4L17#12. | 0.82 | 0.36 |
| KP4L18 | 52 seeds  32:20 | Moderate to poor growth, slight inhibition of root growth. |  | 5.02 | 0.024 |
